# Engaging im/migrant communities in cross-sectoral health and immigration data linkage research.

**DOI:** 10.23889/ijpds.v7i3.1879

**Published:** 2022-08-25

**Authors:** Ruth Lavergne, Ruth Carillo, Shira Goldenberg, Selamawit Hagos, Stefanie Machado, Sandra Peterson, Elmira Tayyar, Padmini Thakore, Cecilia Sierra-Heredia, Susitha Wanigaratne, Mei-Ling Wiedmeyer

**Affiliations:** 1 Dalhousie University; 2 Centre for Gender and Sexual Health Equity; 3 University of California, San Diego and San Diego State University; 4 University of British Columbia; 5 Simon Fraser University; 6 The Hospital for Sick Children Research Institute

## Objectives

We aim to respond to health care barriers experienced by immigrant and migrant (im/migrant) communities through community-engaged research using population-based multi-sectoral linked health and immigration data, alongside qualitative methods. We describe lessons learned with respect to analytic choices and interpretation of findings from data linkage research.

## Approach

We linked Canadian federal immigration data and health data from the province of British Columbia to analyze access to health care services during the COVID-19 pandemic. Immigration data include date and class of arrival, level of education, language ability at arrival, countries of birth and origin, and other personal characteristics. Provinces also collect documentation of immigration status as part of ascertaining health insurance eligibility, data not previously used for research. Planning and carrying out this analysis involved people who come from different countries and have different immigration journeys, such as people with precarious im/migration status, refugees, workers and students.

## Results

Findings underscore that care should be taken in choosing categories to group people using administrative immigration systems data that are relevant to research questions, considering class of arrival, current status, time since arrival, and language ability, alongside intersecting characteristics. In studying COVID-19 infection and access to care, current status (temporary or permanent) was particularly important, as this is tied to both workplace protections/risks and access to care. Time since arrival in Canada and language ability were important in examining questions related to health system navigation, including access to virtual and in-person care. Immigration information recorded at time of registration for provincial insurance offers a new opportunity to include immigration data in analysis, and is particularly helpful in studying impacts of temporary status.

## Conclusion and Relevance

A strength of linked immigration data is that it directly captures administrative categories that are modifiable and that structurally determine health. In interpreting analysis we must emphasize that immigration records and class captured at time of registration for health insurance reflect administratively imposed categories, but may not reflect identities.

